# PM_2.5 _metal exposures and nocturnal heart rate variability: a panel study of boilermaker construction workers

**DOI:** 10.1186/1476-069X-7-36

**Published:** 2008-07-09

**Authors:** Jennifer M Cavallari, Ellen A Eisen, Shona C Fang, Joel Schwartz, Russ Hauser, Robert F Herrick, David C Christiani

**Affiliations:** 1 Department of Environmental Health, Harvard School of Public Health, 665 Huntington Ave, Boston, MA, 02115, USA; 2 Pulmonary and Critical Care Unit, Department of Medicine, Massachusetts General Hospital, 55 Fruit St, Boston, MA, 02114, USA

## Abstract

**Background:**

To better understand the mechanism(s) of particulate matter (PM) associated cardiovascular effects, research priorities include identifying the responsible PM characteristics. Evidence suggests that metals play a role in the cardiotoxicity of fine PM (PM_2.5_) and in exposure-related decreases in heart rate variability (HRV). We examined the association between daytime exposure to the metal content of PM_2.5 _and night HRV in a panel study of boilermaker construction workers exposed to metal-rich welding fumes.

**Methods:**

Twenty-six male workers were monitored by ambulatory electrocardiogram (ECG) on a workday while exposed to welding fume and a non-workday (baseline). From the ECG, rMSSD (square root of the mean squared differences of successive intervals) was summarized over the night (0:00–7:00). Workday, gravimetric PM_2.5 _samples were analyzed by x-ray fluorescence to determine metal content. We used linear mixed effects models to assess the associations between night rMSSD and PM_2.5 _metal exposures both with and without adjustment for total PM_2.5_. Matched ECG measurements from the non-workday were used to control for individual cardiac risk factors and models were also adjusted for smoking status. To address collinearity between PM_2.5 _and metal content, we used a two-step approach that treated the residuals from linear regression models of each metal on PM_2.5 _as surrogates for the differential effects of metal exposures in models for night rMSSD.

**Results:**

The median PM_2.5 _exposure was 650 μg/m^3^; median metal exposures for iron, manganese, aluminum, copper, zinc, chromium, lead, and nickel ranged from 226 μg/m^3 ^to non-detectable. We found inverse linear associations in exposure-response models with increased metal exposures associated with decreased night rMSSD. A statistically significant association for manganese was observed, with a decline of 0.130 msec (95% CI: -0.162, -0.098) in night rMSSD for every 1 μg/m^3 ^increase in manganese. However, even after adjusting for individual metals, increases in total PM_2.5 _exposures were associated with declines in night rMSSD.

**Conclusion:**

These results support the cardiotoxicity of PM_2.5 _metal exposures, specifically manganese. However the metal component alone did not account for the observed declines in night HRV. Therefore, results suggest the importance of other PM elemental components.

## Background

The consistent association between particulate matter (PM) exposures and cardiovascular health effects is well documented [1-3]. As we seek to better understand the mechanisms, research priorities include identifying the PM characteristic(s) responsible for the observed cardiovascular health effects [1,4,5]. Evidence from epidemiological and toxicological studies suggest that composition may play a role in particle-associated cardiovascular responses [4,5].

To date, the majority of epidemiological studies have characterized PM exposures by mass concentration. PM with an aerodynamic diameter ≤ 2.5 μm (PM_2.5_) is often measured due to its ability to penetrate deep into the alveolar regions of the lung, where it can initiate cardiovascular and other health effects. Some studies have focused on identifying air pollution sources responsible for the observed health effects, and there is growing evidence linking traffic-related particle exposures to cardiovascular responses [6]. However, information is lacking on the specific chemical or class of chemical components associated with adverse cardiovascular health outcomes [5,7]. Through source-related studies and toxicological evidence, particulate matter components including soluble organic compounds and metals, specifically transition metals, have been linked to cardiovascular outcomes [4]. The role of metals is further implicated by a study of air pollution exposures among older men finding that metal processing genes modified cardiovascular autonomic responses to PM_2.5_[8].

Our previous research among a cohort of boilermaker construction workers or "boilermakers" occupationally exposed to high-levels of metal-rich PM is the only epidemiological study [9] that has examined the association between specific particulate matter metal components and cardiovascular outcomes in humans. We examined the association between workday heart rate variability (HRV), a measure of cardiovascular autonomic control, and workday PM_2.5 _metal exposures and found statistically significant increases in the 5-min SDNN (standard deviation of normal-to-normal intervals), averaged over the 8–10 hour workday with personal lead and vanadium exposures [9]. This is contrary to the majority of studies reporting an inverse association between HRV and total PM_2.5 _exposure [1].

Our previous investigation which monitored daytime HRV may not have captured the relevant time-period of response. In a subsequent investigation within this cohort, we observed that as compared to the day, night may be a the more relevant time period to capture workday particle-related changes in cardiovascular autonomic response [10]. We observed an inverse exposure-response relationship between workday PM_2.5 _mass and long-duration night HRV, specifically rMSSD (square root of the mean squared differences of successive intervals) and no associations between day HRV and workday PM_2.5 _mass concentration. However, it remains unclear what role the metal composition plays in the particle-related nocturnal HRV changes that we observed. In the current study we sought to examine the association between the metal components of PM_2.5 _both independently and after adjustment for total PM_2.5 _and rMSSD during the night period in a panel study of boilermakers.

## Methods

### Participant recruitment

The Institutional Review Board at the Harvard School of Public Health approved the study protocol, and informed written consent was obtained from each adult prior to participation. From 1999 to 2006, we recruited 36 boilermakers at an apprentice welding school to participate in extensive ambulatory electrocardiogram (ECG) monitoring over two 24-hour periods on both a workday and a non-workday. In 2004 through 2006, in addition to ECG monitoring, 26 boilermaker welders were monitored for workday PM_2.5_exposures which were then analyzed by x-ray fluorescence (XRF) for elemental content. These boilermakers were invited to participate in the study on multiple occasions over the two-year sampling period; 22 (85%) were monitored on one occasion, 3 (12%) were monitored twice, and one was monitored on three occasions for a total of 31 monitoring occasions. On each monitoring occasion, boilermakers were continuously monitored over both a work and non-work day.

### Data collection

Workday monitoring took place at a union welding school, where boilermakers practiced welding, cutting, and grinding techniques. Boilermakers primarily performed shielded metal arc (stick) and gas metal arc welding (TIG), using base metals of mild steel (manganese alloys) and stainless steel (manganese, chromium, and nickel alloys) with electrodes composed mainly of iron with variable amounts of manganese (1–5%). Plasma arc or acetylene torch cutting and grinding also occurred. In addition to workday monitoring, participants were also continuously monitored over a non-workday when they were not welding, grinding or cutting, to establish baseline night HRV. This occurred within 6 months of workday monitoring, but 81% of the observations occurred within the same week. A questionnaire was used to collect information on medical history, current cardiopulmonary symptoms, medication use, demographics, occupational history, and lifestyle factors including smoking history.

### ECG monitoring and tape processing

Participants were fitted with a standard 5-lead ECG Holter monitor. To facilitate good lead contacts, the participant's skin was shaved, if necessary, cleansed with an alcohol wipe, and slightly abraded. Study staff checked leads at the workplace periodically. Each tape was sent to Raytel Cardiac Services (Haddonfield, NJ) for processing and analysis using a DelMar Avionic (Irvine, CA) Model Strata Scan 563. Only beats with an RR interval between 0.6 and 1.5 sec and an RR ratio of 0.8–1.2 were included in the analysis. Trained technicians, blinded to the work and non-work periods, used standard criteria to accept or reject all normal or abnormal findings. Tapes were analyzed in the time domain, and indexes including the square root of the mean of the sum of the squared differences between adjacent NN intervals (rMSSD), the standard deviation of all NN intervals over the entire period (SDNN), and the mean of the standard deviations of all NN intervals for all 5-min segments (SDNN_i_) were calculated over the 7-hr night period (00:00 to 07:00). The current analysis is restricted to the rMSSD measure, which based on our previous analysis, best captures cardiovascular autonomic changes following PM_2.5 _exposure [10].

### Particulate matter exposure assessment and analysis

Personal, integrated, gravimetric samples were collected over the duration of the work shift using a KTL cyclone (GK2.05SH, BGI Incorporated, Waltham, MA) with an aerodynamic diameter cutpoint of 2.5 μm used in line with a pump drawing 3.5 L/min of air. The cyclone was secured to the participant's shoulder in the breathing zone area, and the pump was placed in a padded pouch that was carried by the participant for the entire workday. Each cyclone was fitted with a cassette holding a 37 mm polytetrafluoroethylene membrane filter (Gelman Laboratories, Ann Arbor, MI). The filters were weighed in a temperature- and humidity-controlled room using a standard protocol before and after sampling on an MT5 micro-balance from Mettler-Toledo Incorporated (Columbus, OH). We divided the blank corrected mass of each sample by the sample air volume to calculate the PM_2.5 _mass concentration.

The filters from the cyclone samples were analyzed for elemental components using XRF. Desert Research Institute (Reno, NV, USA) performed all XRF analyses on the PANanalytical Epsilon 5 Energy Dispersive XRF analyzer (Almelo, the Netherlands) and utilized standard operating procedures including quality control and assurance measures [11,12]. For each element the corresponding limit of detection (LOD) was reported as μg of element per filter. Samples below the LOD were assigned the value of the LOD. Metal exposures were calculated by dividing the metal mass by the sampled air volume.

### Statistical methods

Paired t-tests were used to compare the mean rMSSD between work and non-work periods. Due to the skewed exposure distributions, exposure medians and interquartile ranges (Q25 – Q75) were calculated for total PM_2.5 _as well as the following metals: aluminum, chromium, copper, iron, lead, manganese, nickel, and zinc. Spearman correlations between PM mass and metal exposures were estimated.

To investigate the association between PM_2.5 _metal exposures and night HRV, we used linear mixed-effects regression models with random intercept for each subject and unstructured covariance. The mixed-effects models allowed us to account for the correlated outcomes among workers who participated on multiple occasions and to control for unmeasured covariates that differ across subjects. Regression models included a continuous covariate for non-work, night rMSSD to control for participant specific risk factors for HRV such as age and health status that don't vary over the time frame of interest. In addition, we adjusted all models for cigarette smoking by including a dichotomous variable representing smoking status at the time of monitoring. Each metal was modeled separately and due to small sample size, multiple metal models were not considered simultaneously. Since metal and total PM mass exposures covaried, we also investigated the effect of each metal, independent of PM_2.5_, by including total PM_2.5 _in the model along with the metals.

As an alternative approach designed to address the collinearity among exposure variables, we also investigated the association between night HRV and PM_2.5 _metal exposures using a two-step residual model. In the first step, each metal was regressed on total PM_2.5 _in a separate linear model, and the residuals were computed for each observation. Residuals are, by definition, the portion of the outcome (metal content) that is uncorrelated with the independent variable, total PM_2.5 _concentration. We therefore considered the residuals to be measures of the independent contribution of each metal and in the second step, treated them as new exposure variables in the regression models for the health outcome. The residual method approach was adapted from energy adjustment techniques used within nutritional epidemiology [13]. All analyses were performed using SAS version 9.1.

## Results

The 26 male boilermakers were monitored over a total of 31 measurement occasions. Their mean age was 43 years, 81% were white and 74% were non-smokers (Table 1). Five reported hypertension and five reported cardiac compromises, including two reports of myocardial infarction, one stent, one murmur and one arrhythmia. Two individuals reported both hypertension and cardiac conditions. Over the 31 measurement occasions, the difference between night rMSSD on workdays as compared to non-workdays was not statistically significant (p = 0.33).

**Table 1:** Study population characteristics for boilermaker participants (n = 26)

Characteristics	Mean ± SD or n (%)
Male	26 (100)
Age (yrs)^a^	43 ± 11
Range	29 – 64
Race	
White	21 (81)
Black	1 (4)
Hispanic	3 (12)
Asian	1(4)
Current Smoker	7 (26)
Hypertensive	5 (20)
Night^b ^rMSSD (msec)	
Workday^c^	30.3 ± 16.0
Non-workday^c^	32.0 ± 16.0

^a^At study entry. ^b^(00:00 to 07:00) ^c^Over 31 measurement occasions.

The median PM_2.5 _exposure of the participants was 649.8 μg/m^3 ^(Table 2). For the 31 exposure measurements, 12 (39%) nickel, 1 (3%) chromium, and 1 (3%) lead sample had concentrations below the LOD. Boilermakers had the highest exposure to iron, with a median concentration of 225.6 μg/m^3^, followed by manganese at 27.22 μg/m^3^. Aluminum, copper, and zinc median exposures ranged from 4.58 – 0.98 μg/m^3^. Chromium, lead, and nickel were present in the lowest median exposures. In addition to the elements presented in Table 2, XRF analysis also revealed the presence of other elements with median exposures of 21.97 μg/m^3 ^for sodium, 20.92 μg/m^3 ^for potassium, 20.34 μg/m^3 ^for silica, 19.43 μg/m^3 ^for calcium, 3.44 μg/m^3 ^for sulfur, and 1.86 μg/m^3 ^for copper as well as other elements at quantities below a median exposure of 1 μg/m^3^.

**Table 2:** Composition and characteristics for personal, workday PM_2.5 _measurements (n = 31)

		Exposure (μg/m^3^)		
PM_2.5 _Component	Below LOD	Mean	Median	Q25 – Q75
Total	0	799.0	649.8	337.0 – 1052.2
Al	0	5.07	4.58	2.67 – 6.28
Cr	1	0.19	0.16	0.08 – 0.24
Cu	0	3.16	1.86	0.79 – 4.67
Fe	0	319.3	225.6	132.2 – 453.0
Mn	0	27.33	27.22	10.23 – 38.62
Ni	12	0.11	0.04	0.003 – 0.15
Pb	1	0.16	0.14	0.08 – 0.22
Zn	0	2.31	0.98	0.37 – 4.50

Each metal exposure was strongly correlated with total PM_2.5_, with Spearman correlation coefficients ranging from 0.53 for zinc to 0.97 for iron (Table 3). Iron, chromium, aluminum and manganese were most highly correlated with PM_2.5 _(0.97 – 0.91), followed by copper (0.84). The other statistically significant correlations with PM_2.5 _were for lead (0.70), nickel (0.63) and zinc (0.53). The correlations amongst the metals were similarly positive and strong.

**Table 3:** Spearman correlation coefficients within and between PM_2.5 _metal exposures and total PM_2.5 _exposure

	Al	Cr	Cu	Fe	Mn	Ni	Pb	Zn
PM_2.5_	0.91	0.91	0.84	0.97	0.95	0.63	0.70	0.53
Al	1.00	0.91	0.67	0.82	0.89	0.40	0.66	0.50
Cr		1.00	0.74	0.85	0.85	0.52	0.73	0.48
Cu			1.00	0.86	0.73	0.82	0.64	0.56
Fe				1.00	0.91	0.69	0.65	0.50
Mn					1.00	0.55	0.63	0.46
Ni						1.00	0.49	0.40
Pb							1.00	0.50

Mixed model regression analyses for total PM_2.5 _exposures were consistent with our previous findings [10]; after adjusting for non-work night rMSSD, total PM_2.5 _exposure was associated with a statistically significant (p < 0.05) decline in night rMSSD; (-0.006 msec/μg/m^3^, 95% CI: -0.008, -0.003). In separate regression models, increases in each metal exposure were associated with declines in night rMSSD (Table 4, Model 1). In order to try to apportion the association with PM_2.5 _to one or more of the metal components, we needed to adjust for PM_2.5 _in each of the models. Collinearity precludes any useful interpretation of the regression coefficients in a model that includes PM_2.5 _as well as a metal component (Table 4, Model 2). To address this collinearity problem, we used residual models to further explore the association with PM_2.5 _by evaluating the contribution of each metal component. The residual model has the advantage of providing a measure of metal exposure that is independent of PM_2.5_. Therefore, these models allowed us to adjust for PM_2.5 _in the models for each specific metal component. When each metal was regressed on PM_2.5_, the residuals for each model had a mean of zero with interquartile ranges as presented in Table 5. For the regression models using the residual metal exposures, an inverse linear association was seen between HRV and aluminum, chromium, manganese, and lead (Table 5, Model 3). The largest decline in night rMSSD per 1 μg/m^3 ^increase in metal residual was for chromium followed by lead (Table 5, Model 3), although the confidence intervals were broad for these associations. A smaller effect, yet statistically significant (p < 0.05) association, was observed for manganese (Table 5, Model 3). When we expressed exposure in interquartile ranges, the largest change in night rMSSD was observed for manganese with a -3.56 msec decline per interquartile change in manganese exposure independent of PM_2.5 _mass.

**Table 4:** Associations between night rMSSD and individual PM_2.5 _metal exposures

	Model 1		Model 2			
	Metal		Metal		Particulate	
	β_1_	95% CI	β_1_	95% CI	β_2_	95% CI
Al	-0.642**	(-1.07, -0.209)	-0.138	(-2.22, 1.95)	-0.004	(-0.022, 0.013)
Cr	-12.54*	(-29.38, 4.30)	3.40	(-19.22, 26.02)	-0.006*	(-0.014, 0.001)
Cu	-0.294	(-1.38, 0.786)	0.093	(-0.613, 0.799)	-0.006**	(-0.010, -0.001)
Fe	-0.013**	(-0.023, -0.002)	-0.002	(-0.032, 0.028)	-0.005	(-0.016, 0.007)
Mn	-0.130**	(-0.162, -0.098)	-0.145*	(-0.348, 0.058)	0.001	(-0.009, 0.010)
Ni	-4.76	(-24.69, 15.16)	1.03	(-11.10, 13.16)	-0.006**	(-0.010, -0.001)
Pb	-11.90	(-38.72, 14.92)	-0.545	(-23.61, 22.53)	-0.005**	(-0.010, -0.0004)
Zn	-0.108	(-1.06, 0.849)	0.105	(-0.625, 0.834)	-0.006**	(-0.011, -0.001)

Model 1: mixed effects linear regression models for each individual metal, adjusted for baseline night rMSSD and smoking status. Model 2: mixed effects linear regression models with each individual metal and PM_2.5_, adjusted for baseline night rMSSD and smoking status. Regression coefficients (β) are expressed as change in msec of night rMSSD per 1 μg/m^3 ^increase in exposure after adjusting for baseline HRV, smoking status and with or without adjustment for total PM_2.5_. *p < 0.10 **p < 0.05

**Table 5:** Associations between night rMSSD and residual metal exposures

		Model 3		Model 4			
	Metal Residual	Metal Residual		Metal Residual		PM_2.5_	
	Q25 – Q75	β_1_	(95% CI)	β_1_	(95% CI)	β_2_	(95% CI)
Al	-0.57 – 0.59	-1.15	(-3.04, 0.74)	-0.138	(-2.22, 1.95)	-0.005*	(-0.012, 0.001)
Cr	-0.03 – 0.02	-6.59	(-49.78, 36.59)	3.40	(-19.22, 26.02)	-0.006**	(-0.010, -0.001)
Cu	-1.30 – 0.95	0.388	(-0.679, 1.46)	0.093	(-0.613, 0.799)	-0.005**	(-0.010, – 0.000)
Fe	-41.89 – 34.08	0.014	(-0.022, 0.050)	-0.002	(-0.032, 0.028)	-0.006*	(-0.012, 0.000)
Mn	-6.69 – 7.55	-0.250**	(-0.331, -0.169)	-0.145*	(-0.348, 0.058)	-0.003	(-0.007, 0.002)
Ni	-0.07 – 0.05	4.56	(-15.41, 24.52)	1.03	(-11.10, 13.16)	-0.005**	(-0.010, -0.000)
Pb	-0.05 – 0.02	-1.95	(-37.33, 33.43)	-0.545	(-23.61, 22.53)	-0.005**	(-0.010, -0.001)
Zn	-1.69 – 1.24	0.076	(-0.991, 1.14)	0.105	(-0.625, 0.834)	-0.006**	(-0.010, -0.001)

The metal residuals are from the regression of total PM_2.5 _and each metal component, and represent the variation in metal exposure not due to PM_2.5_. Model 3: mixed effects linear regression models for each individual metal residual, adjusted for baseline night rMSSD and smoking status. Model 4: mixed effects linear regression models with each individual metal residual and PM_2.5_, adjusted for baseline night rMSSD and smoking status. Regression coefficients (β) are expressed as change in msec of night rMSSD (msec) per 1 μg/m^3 ^increase in metal residual after adjusting for baseline HRV, smoking status and with or without adjustment for total PM_2.5_. *p < 0.10 **p < 0.05.

Since the metal residual was no longer correlated with PM_2.5_, we controlled for PM_2.5 _mass and can interpret these results as the effect of increasing metal exposures while holding PM_2.5 _constant. After accounting for PM_2.5 _exposure, the associations between the individual metals and HRV weakened or changed in direction (Table 5, Model 4). However an inverse, linear exposure-response relationship remained for aluminum, manganese, and lead, as is consistent with Model 1, although the confidence intervals of these associations widened. In the residual model, holding each metal exposure constant, we observed consistent declines in HRV with increasing total PM_2.5 _exposure, with the exception of manganese, the associations were statistically significant (Table 5, Model 4).

## Discussion

This study provides valuable information on how the metal content of PM contributes to cardiovascular responses, specifically to changes in cardiovascular autonomic control. Our previous investigation found that PM_2.5 _mass was associated with night HRV. In the current investigation by identifying the elemental components of PM_2.5_, we observed an association between increased individual PM_2.5 _metal exposures, specifically manganese, and declines in night rMSSD. We observed heterogeneous associations across the different metals with statistically significant (p < 0.05), declines in HRV per 1 μg/m^3 ^increase aluminum, iron and manganese.

Since PM_2.5 _and metal exposures were correlated, we confirmed the results with residual models. The residual method of analysis confirmed the manganese effect: a -0.250 (95% CI: -0.331, -0.169) msec change in night rMSSD was observed with every 1 μg/m^3 ^increase in manganese residual, which is manganese independent of PM_2.5 _mass. An inverse exposure-response relationship was also confirmed for aluminum, chromium, and lead exposures. Expressed per increase in interquartile range of exposure, the largest decline in HRV was seen for manganese. Since the metal residual was no longer correlated with PM_2.5_, we were able to explore the effect of increasing metal exposures while holding PM_2.5 _constant by adjusting for PM_2.5 _in the metal residual models. We found that after adjusting for PM_2.5_, an inverse relationship, similar in magnitude, persisted for residual manganese, although the confidence intervals were broad.

The association between total PM_2.5 _exposures and night HRV observed in this study is consistent with our previous report among this population [10]. As we hypothesized, unlike the study by Magari et al. [9] that evaluated daytime HRV over work, this study used the susceptible night period and we observed a negative association between night HRV and PM_2.5 _metal exposures. However, a study of highway patrol troopers observed a positive association with increases in post-shift HRV with a source factor related to speed-changing traffic which was dominated by copper, aldehyde and sulfur content of PM_2.5 _exposures [14]. In both our previous investigation and the patrol trooper study the timing of HRV response occurs during or immediately post-exposure; unlike the current study, where HRV was measured in the evening following workday exposure. These disparate results may be capturing the varying time-course of metal responses or the complex interplay between different metals, which is supported by toxicological studies.

While the exact mechanism is unknown, once transition metal components of PM_2.5 _are delivered to the airways, they may catalyze the production of reactive oxygen species (ROS), which cause airway injury and inflammation and start a cascade of pulmonary and cardiac responses [15]. This proposed mechanism is supported in part by *in vitro *and *in vivo *studies showing an association between transition metal exposures and generation of ROS [16], and an association between transition metal exposures and cardiovascular autonomic changes in mice [17] and rats [18] yet not dogs [19]. Alternately, other metals such as zinc has been shown to trigger effects by directly interacting with cellular proteins [20,21].

Results from our current study signal the cardiotoxicity of manganese. Both the standard and residual models presented an inverse exposure-response relationship for manganese and effects persisted after adjustment for total PM_2.5 _exposure suggesting that manganese effects are independent of total fine particulate matter effects. While the cardiotoxicity of manganese has not been previously reported, manganese neurotoxicities are well recognized, and both epidemiological and toxicological studies suggest that manganese exposure may also lead to cardiovascular toxicities [22]. Abnormal ECG parameters, including sinus arrhythmia and ST-T changes, are increased in workers exposed to manganese oxide as compared to controls [22] and Barrington *et al*.[23] report decreased autonomic function and low 24-hr HRV, in manganese alloy workers, though this association has yet to be corroborated by *in vivo *studies, as no significant HRV changes in dogs were observed subsequent to short-term inhalation of aerosol manganese [19].

Yet, metal content does not completely explain the declines in HRV observed within this cohort. An inverse association was observed with PM_2.5 _in the residual models adjusted for individual metals, although PM_2.5 _was not significant in the manganese model. For these metals, this suggests that when the metal content is held constant, there remains a total PM_2.5 _exposure effect. We were unable to determine which, if any, chemical component this is due to. However, the non-metal components may also play a role in the observed cardiotoxicity of PM_2.5 _exposures. In fact, we investigated the association between PM_2.5 _silicon exposures and night rMSSD and observed a -0.17 (95% CI: -0.24, -0.10) msec change per 1 μg/m^3 ^increase in silicon PM_2.5 _exposure after adjusting for non-work HRV and smoking status. The silicon association was attenuated after controlling for PM_2.5 _mass; -0.10 (95% CI: -0.53, 0.33). Due to limitations in the XRF analysis technique, we were unable to measure the silicon complex within the welding fume, however previous investigation of welding fume exposure suggests that silicon is likely in the silica state [24]. Decreases in HRV with silica exposures have been previously observed in a study of aged, ApoE knockout transgenic mice where both ambient particulate matter or silica produced decreases in HRV parameters [25]. The health effects of occupational silica exposure are well recognized; both acute and chronic silica exposures, in the form of crystalline silica, can lead to silicosis, a debilitating fibrotic disease of the lung. Silica exposure has been linked to inflammation and autoimmune diseases [26]. The cardio-toxicity of silica exposure warrants further research.

While we observed signals of cardiotoxicity for manganese, we did not observe an effect for nickel, which has previously been implicated in changes in heart rate and HRV [17]. It is unlikely that one component of PM is responsible for all cardiovascular effects and it is unclear what role the composition mixtures play. One major limitation of this study is the exposure source, which differs substantially from ambient PM_2.5 _or other sources of PM_2.5_. While welding fumes are enhanced in some metals, they may have lower than average proportions of other metals, which may be relevant for other exposure scenarios, and may explain differences in study results. Therefore, the generalizability of the current study results to ambient air pollution exposures is limited.

This study is strengthened by detailed exposure assessment with personal PM_2.5 _exposure monitoring with elemental determination. In total, the elemental mass determined by XRF analysis accounted for 56% of the sample mass. However, one limitation of XRF analysis is that it cannot discern metal oxidation states or identify metals complexes. While the welding fume metals are likely present as metal oxides, the solubility of each element, which may affect toxicity, remains unknown.

While the number of participants available for our study was small, the efficiency of the study was improved by the use of non-work monitoring. Each participant served as his own control by providing baseline, non-work HRV measures over the same time period as work measures. Baseline measures controlled for potential confounding by time-invariant, individual factors such as smoking, health status or general physical activity levels. While particle metal exposures were not monitored during non-work, as compared to workday welding fume exposures, the non-workday ambient metal exposures are likely to be negligible. This study is also strengthened by having separated the exposure period (occurring during work) and the outcome period (occurring in the evening after work) by time, which limits the potential for confounding. In addition to being time-varying, potential confounders must also be associated with exposure during the work time period and be predictors of HRV in the time following exposure. For example, while ventilation during work may be associated with exposure during the work period, it is not associated with night HRV several hours after exposure occurs. Although we were unable to account for predictors of HRV that might vary between the work and non-work periods, such as quantity of caffeine, alcohol and cigarette consumption, for the exposure-response analysis, we hypothesize that neither caffeine nor alcohol consumption were correlated with work PM_2.5 _exposure; therefore the potential for confounding bias by these factors is small. We adjusted for smoking by using a binary variable, but were unable to account for the quantity of cigarettes consumed. However, workers were allowed to smoke on the job, and it is unlikely that the quantity of cigarettes consumed differed between work and non-work periods. Semi-quantitative urinary cotinine levels obtained from NicAlert Strips [27] on a sub-set of the smokers confirmed that there was no difference between work and non-work smoking intensity. Thus, the likelihood of confounding by smoking is low. While the majority of the participants reported no health compromises, there were 8 who reported either or both hypertension or cardiac compromises and of these 3 individuals reported medication use, which could also vary with time. However, when we restricted the cohort to exclude the individuals reporting medication use, the manganese effect persisted in magnitude and increased in statistical significance. Due to small sample size, we were unable to investigate the potential modifying effects of hypertension or cardiac compromises. Since 81% of the non-workday monitoring occurred within the same week as workday monitoring, we were able to control for longer term time trends such as season.

Nevertheless, while we observed statistically significant results for manganese exposures, the lack of statistical significance in the relationships with other metals may be due, in part, to the small sample size. Lastly, the mechanism of metal-related cardiovascular toxicity may be complex and while we observed signals of toxicity in the night period, it is possible that we did not capture early or later effects.

## Conclusion

In summary, this study supports the cardiotoxicity of the metal component of particulate matter exposures. There appears to be a difference in cardiac autonomic response among the metals with a consistent exposure-response relationship observed for manganese. Yet, results suggest the importance of other metal and non-metal particulate matter components in the observed cardiovascular autonomic effects.

## Competing interests

The authors declare that they have no competing interests.

## Authors' contributions

JMC contributed to study design, data collection, analysis and interpretation of the results, and manuscript preparation. EAE and JS contributed to study design, statistical analyses and critical review of the manuscript. SCF contributed to data collection, interpretation of the results, and critical review of manuscript. RFH and RH contributed to exposure assessment and critical review of the manuscript. DCC contributed to study design and analysis, interpretation of the results, and critical review of the manuscript. All authors read and approved the final manuscript.
